# Seroprevalence of brucellosis in humans with non-specific clinical symptoms in Punjab, India

**DOI:** 10.14202/vetworld.2025.819-826

**Published:** 2025-04-19

**Authors:** Deepali Gopal Kalambhe, Brindha Sundar, Jasbir Singh Bedi

**Affiliations:** Department of Veterinary Public Healths and Epidemiology, Centre for One Health, Guru Angad Dev Veterinary and Animal Sciences University, Ludhiana, Punjab, India

**Keywords:** brucellosis, India, non-specific symptoms, Punjab, rose bengal plate test, seroprevalence, standard tube agglutination test, zoonosis

## Abstract

**Background and Aim:**

Brucellosis is a zoonotic disease that remains underdiagnosed in humans due to its non-specific clinical presentations. Punjab, India, is particularly vulnerable to brucellosis due to its high-density livestock farming. This study aimed to estimate the seroprevalence of brucellosis in individuals presenting with non-specific clinical symptoms.

**Materials and Methods:**

A cross-sectional study was conducted at the Centre for One Health, Guru Angad Dev Veterinary and Animal Sciences University, Ludhiana, from January 2021 to December 2021. A total of 137 serum samples were collected from individuals either self-referred or physician-referred for brucellosis testing. The samples were screened using the rose Bengal plate test (RBPT) and confirmed with the standard tube agglutination test (STAT). Data on demographics, symptoms, and occupational exposure were analyzed using SPSS version 26.0, with statistical significance set at p < 0.05.

**Results:**

Overall, 17.52% (24/137) of individuals tested positive using STAT, with antibody titers ranging from 80 IU/mL to >2560 IU/mL. Seropositivity was higher in males (20.83%) than in females (9.77%). The highest seropositivity (42.9%) was observed in individuals aged 71–80 years. Among symptomatic individuals (n = 92), fever was the most common symptom (n = 79), followed by joint pain (n = 13). However, 15.6% of asymptomatic individuals also tested positive. No significant association was found between symptoms and seropositivity (p > 0.05). In addition, self-referred individuals (24.1%) had a higher seropositivity rate compared to physician-referred cases (12.7%). Among occupationally exposed individuals, veterinary officers showed the highest seropositivity.

**Conclusion:**

The study highlights a considerable seroprevalence of brucellosis among various symptomatic and among asymptomatic individuals. Given its non-specific clinical manifestations, routine serological screening is recommended, especially for high-risk groups. A One Health approach integrating human and animal health surveillance is crucial for effective disease control.

## INTRODUCTION

Brucellosis is a zoonotic infection caused by the bacterial genus *Brucella*, which primarily affects livestock and wildlife but can also be transmitted to humans. It remains a significant public health concern in many parts of the world, particularly in regions where animal husbandry is a major component of the economy and where public health measures are inadequate. Human brucellosis is primarily transmitted through direct contact with infected animals or consumption of contaminated animal products, such as unpasteurized milk or cheese. Globally, brucellosis affects more than half a million people annually, with the highest incidence rates reported in the Mediterranean region, the Middle East, parts of Asia, and Latin America [1]. Despite efforts to control the disease through animal vaccination and public health interventions, brucellosis remains endemic in many low- and middle-income countries, including India, because of challenges in implementing effective control measures. In these regions, the disease poses a dual threat to human health and economic stability by affecting livestock productivity and livelihoods. In India, brucellosis is an emerging public health issue, Berhanu and Pal [2] have highlighted its prevalence in humans and animals. In India, brucellosis affects livestock populations and is also an important occupational hazard for humans associated with livestock-related activities, such as veterinarians, animal handlers, slaughterhouse workers, farmers, and laboratory personnel, who are commonly more exposed to animals [2–4]. Approximately 80% of Indians live in close contact with domestic or wild animals because of their occupation, particularly those involved in agriculture, putting them at risk for brucellosis [5, 6].

The state of Punjab, known for its extensive dairy farming, is particularly vulnerable to brucellosis because of the close interaction between humans and livestock. Livestock farming is a crucial component of Punjab’s economy, and the high density of cattle and buffaloes increases the risk of disease transmission to humans. Holt *et al*. [7] have indicated a significant seroprevalence of brucellosis among livestock in Punjab, raising concerns about potential spillover to the human population. Human brucellosis has several clinical manifestations, which often make its diagnosis challenging. The disease is characterized by non-specific symptoms, such as fever, malaise, sweats, fatigue, and joint pain, which can lead to misdiagnosis or delayed diagnosis. In particular, fever of unknown origin is a common presentation that can complicate clinical evaluation [2]. Given its non-specific nature, identifying brucellosis as a causative agent requires thorough clinical evaluation and appropriate serological testing. The variability in symptoms and the lack of specific clinical features necessitate a high index of suspicion, particularly in endemic areas like Punjab. The diagnosis of brucellosis in humans is primarily based on serological testing, with the most commonly used tests being the Rose Bengal test, standard tube agglutination test (STAT), and enzyme-linked immunosorbent assays (ELISA) [5]. These tests detect antibodies against *Brucella* spp. in the patient’s serum, indicating exposure to the bacteria. However, cross-reactivity with other bacteria and variability in test sensitivity and specificity can complicate the diagnostic process [1].

The One Health approach emphasizes the interconnectedness of human beings, animals, and environmental health, advocating for integrated efforts to tackle zoonotic diseases like brucellosis. This approach is particularly relevant in Punjab, where human and animal interactions are frequent and intense, thereby increasing the risk of disease transmission. By adopting a One Health perspective, health professionals can improve disease surveillance, enhance diagnostic capabilities, and implement more effective control measures [8].

Despite the increasing recognition of brucellosis as a significant zoonotic disease, its true burden among individuals presenting with non-specific clinical symptoms remains poorly understood, particularly in endemic regions such as Punjab, India. Existing studies have predominantly focused on high-risk occupational groups or seroprevalence in livestock, leaving a gap in understanding the extent of undiagnosed or misdiagnosed cases among the general population. Moreover, the lack of systematic screening and the non-specific nature of brucellosis symptoms contribute to its underreporting, delaying timely diagnosis and intervention. This study seeks to address this gap by investigating the seroprevalence of brucellosis among individuals with non-specific clinical manifestations, thereby contributing to a more comprehensive understanding of the disease’s epidemiology in human populations.

This study aims to estimate the seroprevalence of brucellosis among individuals presenting with non-specific clinical symptoms in Punjab, India. By employing serological tests, such as the Rose Bengal plate test (RBPT) and the STAT, the study seeks to identify the proportion of individuals with *Brucella*-specific antibodies and assess potential associations with demographic and occupational factors. The findings will contribute to improved diagnostic strategies and public health interventions to enhance disease surveillance and control measures in endemic regions.

## MATERIALS AND METHODS

### Ethical approval and Informed consent

Blood samples were collected from individuals either self-referred or physician-referred for brucellosis testing. Samples were collected by a physician. Written informed consent was obtained from all participants. All data were anonymized to protect patient information.

### Study period and location

This study was conducted from January 2021 to December 2021. This study employed a cross-sectional design to estimate the seroprevalence of anti-*Brucella* antibodies in patients with non-specific clinical symptoms at Center for One Health, Guru Angad Dev Veterinary and Animal Sciences University, Ludhiana Punjab, India.

### Study population

The study population consisted of patients visiting the Center for One Health and presented with non-specific clinical symptoms with no identified cause after initial medical evaluation. Patients included in this study were either referred by a physician from a medical institute or were self-aware of brucellosis testing at this center.

### Sample collection

This study included 137 samples from patients visiting this center for brucellosis testing over a period of 1 year, from January to December 2021. Patients were administered a structured questionnaire that collected demographic and medical information, including occupation and whether or not they were referred to the One Health Center for brucellosis screening. For serological investigation, blood samples (5 mL) were collected from each participant under aseptic conditions in clot activator blood collection tubes. The serum was separated and immediately processed for the RBPT and STAT. The remaining quantities of serum samples were stored at –20°C.

### Serological testing

RBPT and STAT were performed as described by Alton [9], which is recommended by the World Organization for Animal Health (WOAH).

#### RBPT

The RBPT was performed using *Brucella*-colored antigens procured from the Punjab Veterinary Vaccine Institute in Ludhiana. Briefly, the serum samples and RBPT antigen were brought to room temperature (22°C ± 4°C). Equal volumes (25–30 μL) of serum and RBPT antigen were added on a clean grease-free glass slide. The antigen and serum were first thoroughly mixed with a sterile microtip and then by gently rotating the slide in a circular motion. The slide was observed for agglutination for a maximum of 4 min. The development of visible agglutination reaction within 4 min was considered a positive reaction.

#### STAT

STAT was performed using *Brucella* *abortus* S-99 plain antigen (Punjab Veterinary Vaccine Institute, Ludhiana, Punjab). The standard protocol described by Alton [9] was adopted for the testing of human samples. Briefly, six clean and sterile Wasserman’s tubes were arranged in a test tube rack for testing each patient’s samples along with known positive control and no serum negative control. All tubes were properly labeled with patient ID and tube serial number. In the first tube (Sr. No. 1), 0.8 mL, while in the rest of the tubes (Sr. No. 2–6), 0.5 mL of 0.5% carbol-saline was added. The serum was diluted by adding 0.2 mL of the serum sample to the 1^st^ tube, and the contents were mixed thoroughly. The serial dilution of serum was performed by drawing 0.5 mL of the diluted serum sample from 1^st^ tube and transferred it to 2^nd^ tube. Similarly, subsequent dilutions were performed by drawing 0.5 mL of the diluted serum sample from the previous tube and transferred it to the next tube. The 0.5 mL diluted serum from the last tube was discarded. Further, 0.5 mL of *B*. *abortus* S-99 plain antigen was added to each tube, and the mixture was mixed properly. The tubes with antigen-serum (antibody) mixture were incubated in a water bath at 37°C for at least 18 h. After incubation, each tube was observed for agglutination reaction (mat formation). Tubes with mat formation were considered positive, while those with button formation were considered negative. The titer of the sample was calculated using the following formula:


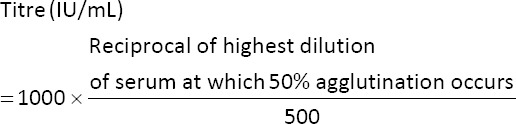


A titer of 160 IU/mL or higher was considered indicative of human brucellosis, as per the national guidelines for endemic regions [10]. Patients with titer of 80 IU/mL and symptoms suggestive of brucellosis were considered doubtful and were recommended for repeat testing after 21 days. However, repeat testing results were not included in the current study. In contrast, those with titer <40 IU/mL were considered negative (in the absence of active symptoms).

### Statistical analysis

All collected data were entered into a compute-rized database and analyzed using IBM Statistical Package for the Social Sciences Statistics version 26.0 (IBM Corp., NY, USA). Descriptive statistics, including frequency distributions and percentages, were used to summarize demographic characteristics, seropositivity rates, and clinical symptoms.

For univariate analysis, Chi-square tests (χ²) were employed to evaluate associations between brucellosis seropositivity (based on STAT results) and categorical variables, such as age groups, gender, referral source, and presence of clinical symptoms. A p < 0.05 was considered statistically significant. The sensitivity and specificity of RBPT were compared to STAT results to determine the agreement between the two serological tests. All statistical tests were conducted at a 95% confidence level, and results were presented in tabular format for clarity.

## RESULTS

Of the total 137 human sera samples screened by RBPT and STAT, evidence of anti-*Brucella* antibodies was observed in 28 (20.44%) individuals by RBPT, while slightly lower seropositivity 24 (17.52%) was detected using STAT (Table 1) with antibody titer ranged between 80 IU/mL and 2560 IU/mL (Table 2). The comparative assessment of the RBPT and STAT results revealed that 24 individuals were positive by both tests, whereas 106 individuals were negative by both tests. Three RBPT-negative serum samples and one RBPT doubtful serum sample showed anti-*Brucella* antibodies titer of 80 IU/mL and 160 IU/mL, respectively, in STAT. However, considering the endemicity of brucellosis in Punjab, the STAT titer of 80 IU/mL or less was considered negative. In statistical analysis, the sera samples positive or negative in both tests were finally considered positive or negative, respectively. The comparative result of both tests is detailed in Table 2.

**Table 1:** Serological tests for the detection of anti-*Brucella* antibodies.

Tests	Serological test			

	RBPT		STAT	
	
	Positive	Negative	Positive	Negative
Number	28*	109	24	113
Percentage	20.44	79.56	17.52	82.5

* Includes doubtful samples that were negative in STAT (titer 80 IU/mL). STAT=Standard tube agglutination test, RBPT=Rose Bengal plate test

**Table 2:** STAT titer for anti-*Brucella* antibodies in tested samples.

RBPT	STAT titer	Frequency of samples with agreement between tests	Final result
Positive	>2560 IU/mL	1	Positive 24
Positive	2560 IU/mL	1	
Positive	1280 IU/mL	4	
Positive	640 IU/mL	5	
Positive	320 IU/mL	7	
Positive	160 IU/mL	5	
Doubtful	160 IU/mL	1	
Positive	80 IU/mL	4	Negative 113
Negative	80 IU/mL	3	
Negative	Negative	106	
Total		137	137

STAT=Standard tube agglutination test, RBPT=Rose Bengal plate test

Assessment of the serological status of brucellosis based on sex revealed that out of 137 participants, 41 were female and 96 were male. The seropositivity of males (20.83% [20/96]) was higher than that of females (9.77% [4/41]) by both tests (Table 3). The highest STAT titer in males was >2560 IU/mL, whereas the highest titer in females was 1280 IU/mL. The frequency of distribution of STAT titer in males and females is tabulated in Table 4.

**Table 3:** Gender-wise seropositivity for brucellosis according to RBPT and STAT.

Gender	Final results		Total

	Negative	Positive	
Female	37	4	41
Male	76	20	96
Total	113	24	137

RBPT=Rose Bengal plate test, STAT=Standard tube agglutination test

**Table 4:** Frequency distribution of STAT titers in male and female patients.

Total (137)	RBPT (%)		STAT (%)		Anti-*Brucella* antibody titer in STAT						
		
	+Ve (28)	-Ve (109)	+Ve (24)	-Ve (113)	>2560 IU/mL	2560 IU/mL	1280 IU/mL	640 IU/mL	320 IU/mL	160 IU/mL	80 IU/mL
Female (41)	14.6 (6)	85.4 (35)	9.7 (4)	90.2 (37)	0	0	1	1	1	1	3
Male (96)	22.9 (22)	77.1 (74)	20.8 (20)	79.2 (76)	1	1	3	4	6	5	2

RBPT=Rose Bengal plate test, STAT=Standard tube agglutination test

The Chi-square test results indicated no statistically significant association between gender and the test results (Table 5).

**Table 5:** Univariate analysis of risk factors associated with brucellosis.

Parameters	Chi-square	p-value
Gender	1.437	0.231
Age group	9.797	0.133
Referral source	2.339	0.126
The presence of symptoms	0.326	0.568
Previously positive for brucellosis	0.453	0.501
Fever	2.574	0.109
Joint pain	0.224	0.636
Back pain	0.685	0.408
Headache	0.225	0.635

A titer of 80 IU/mL was considered negative. Furthermore, in this study, the serological investigation of brucellosis was performed in 116/137 individuals divided into seven age groups. The age of the 21 participants was missing. This investigation detected maximum seropositivity of 42.9% (3/7) in individuals belonging to outliners age group 7 (71–80 years), and 100% sero-negativity was reported in age group 1 (10–20 years) and age group 6 (61–70 years), as given in Table 6.

**Table 6:** *Brucella* seropositivity in different age groups.

Age group	Age range (years)	Number of individuals tested	Number of positive individuals	Percentage positivity
1	10–20	5	All negative	Nil
2	21–30	32	6	18.8
3	31–40	32	9	28.1
4	41–50	17	1	5.88
5	51–60	13	1	7.7
6	61–70	10	All negative	Nil
7	71–80	7	3	42.9
Total		116	20	

The Chi-square test results indicated no statistically significant association between age group and test results (Table 5).

Further analysis by age group revealed a variation in the frequency distribution of STAT titers among different age groups. The oldest individuals grouped in group 7 reported the highest antibody titer of >2560 IU/mL, whereas the adults between 31 and 40 years of age showed an array of titers from 80 to 2560 IU/mL (Table 7).

**Table 7:** Titer of anti-*Brucella* antibody in STAT test in different age groups.

Age group	Age range (years)	Anti-*Brucella* antibody titer						

		>2560 IU/mL	2560 IU/mL	1280 IU/mL	640 IU/mL	320 IU/mL	160 IU/mL	80 IU/mL
2	21–30	0	0	1	1	0	4	1
3	31–40	0	1	1	2	3	2	1
4	41–50	0	0	0	0	1	0	1
5	51–60	0	0	0	1	0	0	0
7	71–80	1	0	1	0	1	0	0
Total		1	1	3	4	5	6	3

STAT=Standard tube agglutination test

Out of 137 individuals, 79 cases of pyrexia of unknown origin were referred by physicians from the Medical Institute, Ludhiana, while 58 individuals were self-aware of brucellosis or were informed by their friends and relatives. Out of 79 cases referred by physicians, 17.7% (14/79) and 12.7% (10/79) cases showed evidence of anti-*Brucella* antibodies using RBPT and STAT tests, respectively, whereas 24.1% (14/58) self-referred individuals were seropositive for brucellosis using both RBPT and STAT (Table 8).

**Table 8:** Sero-positivity of brucellosis in individuals based on their reference.

Total (137)	RBPT (%)		STAT (%)		Anti-*Brucella* antibody titer in STAT						
		
	+Ve (28)	−Ve (109)	+Ve (24)	−Ve (113)	>2560 IU/mL	2560 IU/mL	1280 IU/mL	640 IU/mL	320 IU/mL	160 IU/mL	80 IU/mL
DMC (79)	17.7 (14)	82.3 (65)	12.7 (10)	87.3 (69)	0	0	2	3	3	2	6
Self (58)	24.1 (14)	75.9 (44)	24.1 (14)	75.9 (44)	1	1	2	2	4	4	0

*Titer of 80 IU/mL was considered negative. RBPT=Rose Bengal plate test, STAT=Standard tube agglutination test

The Chi-square test results indicate no statistically significant association between referral source and test results (Table 5).

Analysis of the frequency of symptoms showed that 54/137 individuals had no symptoms while 92 individuals had symptoms. The frequency distribution of various symptoms is tabulated in Table 5. Regarding fever, 58 participants reported no fever, with 7 testing positive (12.1%), while 79 participants reported fever, with 18 testing positive (22.8%). For the presence of any symptoms, 45 participants reported no symptoms, with 7 testing positive (15.6%), while out of 92 participants with symptoms, 18 tested positive (19.6%). Regarding previous test results, 135 participants were previously negative, with 25 testing positive (18.5%), while 2 participants were previously positive, both testing negative (100%). For joint pain, 124 participants reported no joint pain, with 22 testing positive (17.7%), while 13 participants reported joint pain, with 3 testing positive (23.1%). Regarding back pain, 134 participants reported no back pain, with 109 testing negative (81.3%) and 25 testing positive (18.7%), while 3 participants reported back pain, all testing negative (100%). Regarding headache, 136 participants reported no headache, with 111 testing negative (81.6%) and 25 testing positive (18.4%), while 1 participant reported headache, testing negative (100%) (Table 9). No significant difference was observed between symptoms and seropositivity for brucellosis (Table 5).

**Table 9:** Frequency distribution of various symptoms among patients.

Total (n = 137)	Symptoms (yes) (n = 92)		Symptoms (no) (n = 45)	
		
Clinical presentation	Sero-positive (18)	Sero-negative (74)	Sero-positive (7)	Sero-negative (38)
Fever	18	61	7	51
History of previous positivity	0	2	25	110
Joint pain	3	10	22	102
Back pain	0	3	25	109
Headache	0	1	25	111
Epididymitis/orchitis	0	1	25	111
Other (accidental contact with vaccine, chills, weakness)	0	6	24	107

Furthermore, in this study, 23/137 individuals provided information on occupation. These 23 individuals were veterinary officers (n = 5), veterinary pharmacists (n = 16), and livestock farmers (n = 2). The observation showed that more veterinary officers were seropositive for brucellosis than the other two occupations. The results are summarized in Table 10. However, the highest antibody titer (1280 IU/mL) among the three occupations was reported by two veterinary pharmacists.

**Table 10:** Sero-positivity of brucellosis according to occupation.

Occupation	Number of individuals	Standard tube agglutination test positive	STAT titer (IU/mL)
Veterinary officers	5	4	640 (two) 320 (two)
Veterinary pharmacists	16	3	160 (one) 1280 (two)
Livestock farmer	2	Negative	

STAT=Standard agglutination test

## DISCUSSION

This study estimated the seroprevalence of brucellosis among patients suspected of having non-specific clinical symptoms using the RBPT and standard tube agglutination test. The findings revealed the seroprevalence of brucellosis in patients to be 20.44% and 17.52% by RBPT and STAT, respectively, highlighting the importance of brucellosis as a public health concern in the region.

The consensus seropositivity rate obtained by both STAT and RBPT in this study was 17.52% (24/137). The antibody titer of STAT in this study ranged from a minimum of 80 IU/mL to more than 2560 IU/mL. However, considering the endemicity of brucellosis in Punjab, the titer ≥160 IU/mL was considered positive, whereas a titer of 80 IU/mL was recorded as negative. The individuals were advised to repeat testing after 21 days. The STAT, which is known for its high specificity, is widely used in endemic regions to confirm cases of human brucellosis. However, the RBPT, due to it’s easy of performance and rapid results, remains an effective screening tool, especially in resource-limited settings [8]. The findings of this study suggest that the combination of both tests helps in the accurate diagnosis of brucellosis in humans.

The findings of human seropositivity for brucellosis in this study are higher than those reported in a previous study by Holt *et al*. [7] from Punjab, wherein the authors observed a lower (9.7%) seropositivity in humans having direct contact with livestock populations. The variation in seroprevalence across these two studies from the same region could be attributed to several factors, including differences in sample size, study population, population demographics, and exposure risks. Moreover, the present study focused on brucellosis testing only in individuals who were either self-referred or referred by the Medical Institute, Ludhiana after having persistent non-specific clinical symptoms, and testing results for other potential infections were negative.

The results of the current study align with the findings of another study on occupationally exposed humans such as veterinarians, veterinary pharmacists, and animal handlers [4]. Similar results have been reported in veterinarians and para-veterinarians in Punjab [11]. Taken together, all studies indicate a significant burden of human brucellosis, particularly in high-risk occupations in Punjab.

Few other studies in India have measured the seroprevalence of human brucellosis among occupationally exposed individuals ranging from 2.4%–55.0% [12–16]. The average seroprevalence (28.7%) of all these studies is in agreement with the findings of the present study.

The assessment of brucellosis in the present study revealed that males had higher seropositivity with the highest STAT titer as compared to females, 9.77% (4/41). The findings of this study are similar to those of Gemechu and Gill [11]. However, neither study found a statistical association between sex and brucellosis seropositivity. This may be attributed to the fact that individuals of both genders are equally involved in high-risk activities like livestock rearing, although with variations in specific activities performed based on gender roles [17].

Further, regarding the results of brucellosis seropositivity in different age groups, the highest seropositivity was observed in elderly individuals (71–80 years; 42.9%), followed by the age group of 31–40 years. The lowest seroprevalence was reported in the age group of 10–20 years and 61–70 years. Comparing the results of the present study with those of previous studies conducted in the same region revealed different observations. The previous study by Gemechu and Gill [11] observed the age group of 26–35 years as the most susceptible age group, followed by 46–55 years and 16–25 years. A sero-epidemiological survey conducted in Morocco found a strong direct proportional relationship (p = 0.001) between age and seropositivity, concluding that brucellosis seropositivity increases with increasing age in humans [18]. The increase in seropositivity with age may be attributed to prolonged exposure to risk factors over time, such as continued engagement in livestock handling, consumption of unpasteurized dairy products, or other occupational and environmental exposures that facilitate the transmission of the disease [18].

One of the significant challenges in diagnosing brucellosis is its non-specific clinical presentation, which often leads to underdiagnosis and mismanagement [19]. This study underscores the importance of considering brucellosis for the differential diagnosis of febrile illnesses, particularly in regions where the disease is endemic. Given the zoonotic nature of brucellosis, implementing a One Health approach that promotes collaboration between the human and veterinary health sectors is crucial.

In this study, out of 137 cases, 54 individuals had no symptoms, whereas 92 individuals had symptoms. The most common symptom was fever, followed by joint pain. Of the 45 asymptomatic cases, 15.6% were serologically positive, while 19.6% (18/92) symptomatic cases were also serologically positive. None of the symptoms in the present study were statistically significant for serological positivity for brucellosis. The findings of this study are in agreement with those of a previous study by from the same region in which the authors reported 11.6% seropositivity in asymptomatic cases [11]. The finding of this study are lower than the reported (88.7%) by Mantur *et al*. [20]. Furthermore, the clinical presentations of symptoms such as fever, headache, chills, weakness, back pain, joint pain, and orchitis in the present study are somewhat similar to those reported previously in India [11, 20, 21]. The lack of a significant association between symptoms and brucellosis seropositivity in this study could be due to the non-specific nature of brucellosis symptoms, which often overlap with other febrile illnesses. In addition, a notable proportion of asymptomatic seropositive cases suggest the presence of subclinical infections.

## CONCLUSION

This study highlights a seroprevalence of 17.52% for brucellosis in individuals with non-specific clinical symptoms in Punjab, India, underscoring the underdiagnosed nature of the disease in endemic regions. Among the study participants, male individuals (20.83%) exhibited higher seropositivity compared to females (9.77%), with the highest seropositivity observed in the elderly age group (71–80 years, 42.9%). Fever was the predominant symptom among symptomatic individuals (85.9%), yet no significant association was found between clinical symptoms and seropositivity (p > 0.05), emphasizing the non-specific nature of brucellosis symptoms. In addition, self-referred individuals (24.1%) showed a higher seropositivity rate than physician-referred cases (12.7%), indicating increased disease awareness in some populations. Among occupationally exposed individuals, veterinary officers exhibited the highest seropositivity, reinforcing the occupational risk associated with livestock handling.

This study provides significant insights into the seroprevalence of brucellosis in Punjab, particularly among individuals presenting with unexplained clinical symptoms. A major strength of the study is its focus on a previously underexplored patient group, along with the use of both RBPT and STAT tests to enhance diagnostic accuracy. The inclusion of asymptomatic individuals also sheds light on potential subclinical infections, contributing to the silent spread of the disease. However, the study is limited by its cross-sectional design, which does not allow for causality to be determined, and its relatively small sample size, which may limit the generalizability of the findings. In addition, the study relies solely on serological tests, which, while widely used, may have limitations in sensitivity and specificity. Selection bias is another potential limitation, as the study included only self-referred and physician-referred individuals, possibly overlooking undiagnosed cases in the general population.

Future studies should adopt a longitudinal approach to track disease progression and trends over time. The incorporation of molecular diagnostic techniques such as PCR and ELISA would improve diagnostic accuracy and reduce false positives. Expanding seroprevalence studies to include a wider geographic and occupational population would provide a clearer picture of the true burden of brucellosis. In addition, awareness campaigns targeting high-risk groups, including veterinary professionals and dairy farmers, should be prioritized to facilitate early detection and prevention. Implementing a One Health-based integrated surveillance system, with collaboration between the human health, veterinary, and public health sectors, would be instrumental in curbing the spread of brucellosis and improving public health outcomes in endemic regions.

The findings of this study emphasize the public health significance of brucellosis in Punjab and the need for enhanced diagnostic vigilance, improved surveillance, and targeted interventions. A multisectoral approach, integrating human and veterinary health sectors, is essential to effectively control and prevent brucellosis in endemic regions.

## AUTHORS’ CONTRIBUTIONS

DGK: Contributed to the study’s conception and design, conducted testing, acquired and analyzed data, and drafted the manuscript. BS: Contributed to data analysis, manuscript revision, and critical review. JSB: Supervised the study and provided critical revisions. All authors have reviewed and approved the final manuscript and agree to be accountable for the work.

## References

[1] Hull N.C, Schumaker B.A (2018). Comparisons of brucellosis between human and veterinary medicine. Infect Ecol. Epidemiol.

[2] Berhanu G, Pal M (2021). Brucellosis: A highly infectious zoonosis of public health and economic importance. J. Emerg. Environ. Technol. Health Prot.

[3] Shakuntala I, Milton A.A, Sanjukta R.K, Kakoty K, Karam A, Dutta A, Puro K, Sen A, Das S, Ghatak S (2021). Isolation and sero-genomo-epidemiological studies on *Brucella* infection in dairy cattle in Meghalaya, India. Comp. Immunol. Microbiol. Infect. Dis.

[4] Proch V, Singh B.B, Schemann K, Gill J.P.S, Ward M.P, Dhand N.K (2018). . Risk factors for occupational *Brucella* infection in veterinary personnel in India. Transbound. Emerg. Dis.

[5] Deka R.P, Magnusson U, Grace D, Shome R, Lindahl J.F (2020). Knowledge and practices of dairy farmers relating to brucellosis in urban, peri-urban and rural areas of Assam and Bihar, India. Infect. Ecol. Epidemiol.

[6] Kant N, Kulshreshtha P, Singh R, Mal A, Dwivedi A, Ahuja R, Mehra R, Tehlan M, Ahmed P, Kaushik S, Shipra (2018). A study to identify the practices of the buffalo keepers which inadvertently lead to the spread of brucellosis in Delhi. BMC Vet. Res.

[7] Holt H.R, Bedi J.S, Kaur P, Mangtani P, Sharma N.S, Gill J.P, Singh Y, Kumar R, Kaur M, McGiven J, Guitian J (2021). Epidemiology of brucellosis in cattle and dairy farmers of rural Ludhiana, Punjab. PLoS Negl. Trop. Dis.

[8] Alam A, Sami H, Hashmi S.Z, Gururaj K, Khan M.A, Khan P.A, Ahmad H, Fatima N, Khan H.M (2024). Seroprevalence and risk factor analysis of brucellosis among dairy farmers in Aligarh region, North India: Creating awareness of a neglected disease *Access Microbiol*.

[9] Alton G.G (1988). Techniques for the Brucellosis Laboratory. Institut National de la Recherche Agronomique, Paris.

[10] Andriopoulos P, Tsironi M, Deftereos S, Aessopos A, Assimakopoulos G (2007). Acute brucellosis: Presentation, diagnosis, and treatment of 144 cases. Int. J. Infect. Dis.

[11] Gemechu Y, Gill J.P.S (2011). Seroepidemiological survey of human brucellosis in and around Ludhiana, India. Emerg. Health Threats J.

[12] Mangtani P, Berry I, Beauvais W, Holt H.R, Kulashri A, Bharti S, Sagar V, Nguipdop-Djomo P, Bedi J, Kaur M, Guitian J, McGiven J, Kaur P, Gill J.P.S, Grover G.S, Kumar R (2020). The prevalence and risk factors for human *Brucella* species infection in a cross-sectional survey of a rural population in Punjab, India. Trans. R. Soc. Trop. Med. Hyg.

[13] Shome R, Kalleshamurthy T, Shankaranarayana P.B, Giribattanvar P, Chandrashekar N, Mohandoss N, Shome B.R, Kumar A, Barbuddhe S.B, Rahman H (2017). Prevalence and risk factors of brucellosis among veterinary health care professionals. Pathog. Glob. Health.

[14] Jamir T, Laskar S.A, Sarma V, Deka N.N (2020). Brucellosis in patients with pyrexia of unknown origin in Assam, India: A retrospective record review. Lancet Glob. Health.

[15] Ghugey S.L, Setiam M.S, Deshmukh J.S. (2021). Human brucellosis: Seroprevalence and associated exposure factors among the rural population in Nagpur, Maharashtra. India. J. Fam. Med. Prim. Care.

[16] Kumar K, Patnaik R, Kumari H, Sharma N (2022). Acute febrile illness: A systematic review of infectious aetiologies among patients. J. Pharm. Negat. Results.

[17] Thakur S, Varma S.K, Goldey P.A (2001). Perceptions of drudgery in agricultural and animal husbandry operations: A gender analysis from Haryana State, India. J. Int. Dev.

[18] Faddane K, Moumni H, Cherkaoui I, Lakranbi M, Bourjilate F, Hamdi S, Saile R, El-Azhari M (2022). Seroprevalence of brucellosis among farmers in Morocco. Moroc. J. Public Heath.

[19] Kiambi S.G, Fèvre E.M, Omolo J, Oundo J, de Glanville W.A (2020). 'Risk factors for acute human brucellosis in Ijara, North-Eastern Kenya. PLoS Negl. Trop. Dis.

[20] Mantur B.G, Biradar M.S, Bidri R.C, Mulimani M.S, Veerappa K, Kariholu P, Patil S.B, Mangalgi S.S. (2006). Protean clinical manifestations and diagnostic challenges of human brucellosis in adults: 16 years'experience in an endemic area. J. Med. Microbiol.

[21] Biswal M, Krishnamoorthi S, Bisht K, Sehgal A, Kaur J, Sharma N, Suri V, Sethi S (2020). Rickettsial diseases: Not uncommon causes of acute febrile illness in India. Trop. Med. Infect. Dis.

